# Predicting biomarkers of progressive pulmonary fibrosis: morphological, cytokine profile, and clinical portrait

**DOI:** 10.3389/fimmu.2025.1514439

**Published:** 2025-06-19

**Authors:** Nicol Bernardinello, Federica Pezzuto, Lauren D’Sa, Luca Vedovelli, Chiara Giraudo, Anamaria Chelu, Cecilia de Chellis, Francesca Lunardi, Francesco Fortarezza, Francesca Boscaro, Elisabetta Cocconcelli, Paolo Spagnolo, Elisabetta Balestro, Fiorella Calabrese

**Affiliations:** ^1^ Respiratory Disease Unit, Department of Cardiac, Thoracic, Vascular Sciences and Public Health, University of Padova, Padova, Italy; ^2^ Department of Cardiac, Thoracic, Vascular Science, and Public Health, University of Padova, Padova, Italy; ^3^ Department of Histopathology, Royal Brompton and Harefield Hospitals, Guy’s and St Thomas’ National Health Service (NHS) Foundation Trust, London, United Kingdom; ^4^ Pulmunology Unit, Ospedale Arco di Trento, Trento, Italy

**Keywords:** interstitial lung disease, traction bronchiectasis, IL-9, progressive pulmonary fibrosis, lung fibrosis

## Abstract

**Objective:**

The term progressive pulmonary fibrosis (PPF) refers to a specific disorder that becomes worse despite optimal treatment. The pathogenic explanation of this progressive worsening is still to be found. In this study, we explored whether any histological, molecular, radiological, or clinical features could predict a progressive phenotype in patients with fibrotic interstitial lung diseases.

**Methods:**

Two hundred and fifteen patients with PPF other than idiopathic pulmonary fibrosis (IPF) and connective tissue disease-associated ILD (CTD-ILD) were followed in our ILD clinic between January 2016 and May 2023. Based on tissue block availability, 48 patients were definitively enrolled. Progression was defined according to the most recent guidelines. Clinical, radiological, and functional data were also collected retrospectively and correlated with tissue morphological and molecular cytokine profiles.

**Results:**

Fifteen patients were classified as progressors (PPF) and 33 as non-progressors (nPPF) with similar age at diagnosis and gender. PPF showed a higher prevalence of traction bronchiectasis (80% vs. 27%; p=<0.001) at CT scan and lower functional parameters [FVC: 2.42 L vs. 3.37 L; p=0.004; TLC: 3.83 L vs. 4.65 L; p=0.027] at diagnosis. Lung specimens revealed a significant overexpression of IL9 in the PPF compared to the nPPF group (p=0.049). Boruta algorithm analysis showed that lymphoid aggregates and traction bronchiectasis at diagnosis are the most important variables in determining the PPF status.

**Conclusions:**

The present results increase the understanding of the pathological mechanisms of PPF, offering potential avenues for improved prognostication and therapeutic intervention.

## 1 Introduction

Interstitial lung diseases (ILDs) refer to a wide spectrum of heterogeneous entities characterized by lung scarring and stiffness of the respiratory system (1). In this context, some patients can remain stable over time, whereas some evolve into a progressive phenotype with a prognosis similar to idiopathic pulmonary fibrosis (IPF), the prototype of progressive and deadly fibrosing ILD (2). The prevalence of patients with ILD who develop a progressive phenotype has varied in the last decade and has been reported to be between 13% and 53% (3, 4). Recent guidelines have defined the criteria for progressive pulmonary fibrosis (PPF), delineating a specific group of patients who show a worsening of the functional, radiological, or clinical features of their underlying respiratory condition despite receiving optimal treatment (5).

Currently, the greatest challenge in this field is to find risk factors that can predict the evolution of fibrosis and its progression in patients with different ILDs. Several risk factors that predispose patients to the progression of fibrosis and then death have been reported at diagnosis: these include older age, radiologic usual interstitial pneumoniae (UIP) pattern, extensive traction bronchiectasis at high-resolution computed tomography (HRCT), increased level of monocytes, and short telomere syndrome (6–10). A recent study by Barnett and colleagues tried to combine data from HRCT and bronchoalveolar lavage (BAL) at baseline to predict progression in a retrospective derivation cohort of 240 patients with fibrosing ILD and a validation cohort of 290 patients (7). Notably, they found that BAL lymphocyte proportion, UIP pattern, and a fibrosis extent greater than 20% were significantly and independently associated with disease progression. Combined analyses also showed that BAL lymphocytosis was rare when there was extensive fibrosis on HRCT. In another study by Watase M. et al. (11), multivariate logistic regression analysis revealed that sex (male), age, white blood cell fraction in BAL fluid, neutrophil to lymphocyte ratio (NLR), and CD8+ T cells in BAL fluid were independent diagnostic predictors for PPF. None of the several putative biomarkers associated with a progressive phenotype that have been reported have been validated for clinical use; thus, investigating predictors of progression is still urgently needed to promptly tailor treatment and ameliorate the survival of patients with fibrosing ILD.

In light of these considerations, our study aimed at exploring, in a well-characterized cohort of fibrosing ILD patients, the usefulness of combining clinical features, HRCT, and, for the first time, molecular features that may discriminate at diagnosis of those patients that undergo therapy who are more likely to progress over time compared to those that are more likely to remain stable.

## 2 Methods

### 2.1 Study population and design

In this monocentric study, a total of 215 patients who received the diagnosis of a type of fibrosing ILD were consecutively enrolled between January 2016 and May 2023. All the cases were reviewed by a multidisciplinary team (MDT), and the final diagnosis was obtained according to the most recent guidelines (5). For our purposes, inclusion criteria were i) age > 18 years, ii) a fibrosing ILD diagnosis, iii) the presence of adequate lung tissue obtained at diagnosis, and iv) a clinical-radiological follow-up longer than at least one year. The patients without histological specimens obtained at the time of diagnosis were excluded from the analysis. Furthermore, the patients with non-fibrotic sarcoidosis, cystic disease (such as lymphangioleiomyomatosis, or Langerhans histiocytosis), IPF, and lung involvement associated with connective tissue disease-ILD (CTD-ILD) were also excluded from the study. CTD-ILD was excluded because, in most cases, histological proof is not needed for the diagnosis. The same was made for IPF, because is progressive by definition. All patients were followed at the University Hospital of Padova, and for the entire population, clinical, functional, radiological, and histological data were collected at the time of diagnosis and during follow-up visits, with the exception of the histological findings. The PPF was assessed according to the most recent guidelines (5). In the end, of the initial 215 patients with a diagnosis of fibrosing ILD evaluated in our center, the study included only 48 patients with fibrosing ILD (**Supplementary Figure S1**). HRCT at diagnosis was evaluated by an expert radiologist (CG). The possible presence of honeycombing, traction bronchiectasis, ground glass opacity, consolidation, or reticular abnormalities was assessed and dichotomized for statistical analysis as yes/no. In the cohort studied, nobody developed an autoimmune disease or changed the type of diagnosis during the follow-up. This study was performed following the declaration of Helsinki and was approved by the ethics committee of the University Hospital of Padua (n°428/AO/17). Informed consent was obtained from all patients.

### 2.2 Histological evaluation

Ten patients underwent surgical videothoracoscopy and 38 transbronchial biopsies (TBB) for diagnostic purposes. TBB were considered adequate for the present study when at least six to biopsies were obtained from well-aerated lung parenchyma in more than half of the samples. All tissue samples were stained with hematoxylin and eosin (H&E) and Masson’s trichrome (MT) staining. Sections stained with H&E and MT were digitalized as whole slide images (WSIs) in tiff format at 40× magnification using Aperio CS, Leica Microsystems. Image analysis was performed using QuPath (version 0.4.3), an open-source software that allows for the visualization, annotation, and measurement of histological features in digital slides. Using QuPath software, each image was subjected to automatic correction of the image color scales, through the software’s ‘Estimate stain vectors’ function. The analysis was performed on the entire section of the lung tissue specimen to avoid selection bias. Inflammatory areas were evaluated in the hematoxylin channel and positive immunohistochemical (IHC) areas in the DAB channel, with measurements made at 0.5 μm/pixel. A percentage ratio (RQuPath) was calculated for each case using the formula: RQuPath = Positive Area/Total Area × 100. A trained pathologist assessed the severity and distribution of inflammation by combining digital measurements with morphological interpretation. Inflammatory infiltrates were categorized based on their localization (peribronchial, interstitial, or subpleural) and extent (focal, patchy, or extensive), using both digital analysis and direct visual review. Lymphoid aggregates, follicles with germinal centers, granulomas, and fibroblastic foci were manually counted on H&E-stained slides and expressed as number per square mm of area examined. Pigmented intra-alveolar macrophages were recorded as either present or absent.

### 2.3 Molecular analyses of inflammatory mediators

For molecular analyses of cytokine expression, the RNA was extracted from formalin-fixed paraffin-embedded tissue as previously reported (12). Cytokine gene expression was examined using TaqMan^®^ Array Human Cytokine Network (Applied Biosystems) with predesigned human gene-specific primers and with probes based on published cytokine sequences and following the manufacturer’s instructions. Each resulting solution was then used to load the array plate, with 20 μL of solution dispensed into each well. The Array Human Cytokine Network 96-well Plate, which includes 28 assays targeting cytokine network-associated genes and four assays for candidate endogenous control genes, was used, and all assays were performed in triplicate. This array was selected because it comprises key genes involved in the regulation of immune responses, inflammation, and fibrogenesis, pathways that are central to the pathogenesis of ILDs. In particular, it includes cytokines such as IL-1β, IL-6, TNF-α, and TGF-β, which are widely described in the literature as critical mediators of ILD progression and lung fibrosis (13, 14).

For cytokine genes after amplification, the average cycle threshold (Ct) was determined for each sample. Subsequently, the ΔCt value was calculated by normalizing the expression of target genes with the 18S housekeeping gene. Relative transcript levels (fold-changes) were then calculated using the formula x = 2^(-ΔΔCt), with inflammatory controls (IC) and healthy controls being used for comparative analysis. In the case of non-expression, a ΔCt value of 30 was assigned by convention to allow statistical processing. For the comparison between PF-ILD and nPF-ILD patients, cytokine overexpression was assessed using ΔCt values, with lower ΔCt indicating higher expression. A cytokine was considered overexpressed when the median ΔCt in one group was at least 1.0 cycle lower than in the other group, corresponding approximately to a biologically meaningful (≥2-fold) increase in transcript abundance.

### 2.4 Statistical analysis

Patient characteristics are described using absolute numbers and percentages for categorical variables and median and range for continuous variables. Differences between groups were assessed using the Mann-Whitney U tests. Distributions of categorical variables were investigated by χ2 and the Fisher’s exact test or Pearson chi-square test, as appropriate. Survival curves were performed using Kaplan-Meier analysis. All statistics were calculated using ΔCt values, which are inversely related to the expression value of the target gene. ΔCt values were compared to all available clinical and morphological data. The Kruskal-Wallis rank-sum test was used to compare cytokine expression data as a continuous variable (ΔCt). Several clinical factors were considered as possible confounders for data interpretation and thus were investigated: all demographic and clinical features e.g., age, sex, smoking history, comorbidities, and type of disease. Feature selection was implemented using a machine-learning algorithm based on a random forest (Boruta). The Boruta algorithm is used to identify the most relevant predictors that impact the outcome of interest (in our case, being in the PF-ILD group). Before applying the feature selection algorithm, the dataset was imputed using a random forest-based method. To add robustness to the feature selection analysis, the Boruta algorithm was iterated through five different initial seeds of the random number generator, and the features that were identified as important in all five iterations were finally kept as important. Logistic regression analysis was also performed. P values <0.05 were used as the criterion for statistical significance. All data were analyzed using R (v. 4.3.3) with the {Boruta} and {gtsummary} packages. The survival graphic was performed with Jamovi (Version 2.3.21.0). The full analysis code and all demographic characteristics, and clinical/radiological/pathological original datasets are available at https://researchdata.cab.unipd.it/id/eprint/1329.

## 3 Results

### 3.1 Clinical characteristics of the study population

In our cohort, 15 (31%; F:M 6/9) patients resulted as progressors (PF-ILD) and 33 (69%; F:M 11/33) as non-progressors (nPPF). All patients were naïve from any immunosuppressive/antifibrotic/corticosteroid therapy at the moment of diagnosis/histological examination. After a multidisciplinary team discussion, 17 patients were classified as fibrosing organizing pneumonia (35%), 9 as hypersensitivity pneumonitis (18.75%), 8 as fibrosing nonspecific interstitial pneumonia (NSIP) (16.6%), 7 as smoking-related interstitial lung disease (14.5%), 2 as pleuro-parenchymal fibroelastosis (4.16%), and 5 as an unclassifiable disease (10.4%) even after multiple MDT discussion. In the two groups, age at diagnosis, gender, smoking history, and body mass index were similar between PPF and nPPF (all p=ns). All these data are reported in **Table 1**. Patients with PPF showed lower ten-year survival compared to the nonprogressive group, as reported in **Supplementary Figure S2** (p=0.027).

**Table 1:** Demographics and clinical features of the overall population and patients with PPF and nPPF.

	Overall (48)	PPF (15)	nPPF (33)
Age at diagnosis – years	62.1 (54 - 72)	63 (59 - 66)	62 (54 - 72)
Sex – Male n° (%)	31 (64%)	9 (60%)	22 (67%)
BMI – (Kg/m^2^)	28.63 (24.3 - 34.5)	29.4 (26.6 - 34.5)	28.6 (24.3 - 31.5)
Pack-Years	10 (0 - 26)	17 (8 - 28)	2 (0 - 26)
Current smoker – n°(%)	3 (6.25%)	1 (6.7%)	2 (6.1%)
Former smoker – n°(%)	27 (56%)	11 (73%)	16 (48%)
Comorbidities			
• Cardiovascular – n° (%)	36 (75%)	12 (80%)	24 (73%)
• Metabolic – n° (%)	16 (33%)	6 (40%)	10 (30%)
• GERD – n° (%)	10 (21%)	3 (20%)	7 (21%)
• Respiratory - n° (%)	14 (29%)	3 (20%)	11 (33%)
• Oncological - n° (%)	9 (19%)	2 (13%)	7 (21%)

BMI, Body mass index; GERD, Gastroesophageal reflux disease. To compare demographics between PPF (progressive pulmonary fibrosis) and nPPF (nonprogressive pulmonary fibrosis), the chi-square test and Fisher’s t-test for categorical variables, and Mann–Whitney t-test for continuous variables were used.

### 3.2 Functional and radiological characteristics

At the time of diagnosis, patients with PPF showed higher functional impairment as revealed by FVC and TLC (both p<0.05; **Table 2**). At the last follow-up, lower functional parameters were significantly more evident in patients with PPF [FVC: 2.11 L (1.66 - 2.44) vs. 3.64 L (2.62 - 3.95); p=<0.0001; DLCO: 33% (31 - 52) vs. 63% (53 - 70); p=<0.0001] (**Supplementary Table S2**). Indeed, patients with PF-ILD developed more frequent respiratory failure on effort (p=0.002) and at rest (p=0.003) compared with patients with nPPF, as reported in **Table 1**. At diagnosis, high-resolution CTs showed a higher prevalence of traction bronchiectasis (80% vs. 27%; p=<0.0001), with a lower prevalence of consolidation (13% vs. 42%; p=0.048), in patients with PPF compared to nPPF (**Table 2**). During the course of the disease, the two populations received similar immunosuppressive/corticosteroid treatment. However, antifibrotic therapy was higher in patients with PPF (p=<0.001).

**Table 2:** Main radiological, functional and histological features at diagnosis in patients with PPF (progressive pulmonary fibrosis) and nPPF (nonprogressive pulmonary fibrosis).

	PPF (15)	nPPF (33)	p-value
Functional parameters			
**FVC (L)**	2.42 (1.98 - 2.79)	3.37 (2.54 - 3.92)	**0.004**
**FVC (%)**	79 (67 - 91)	89 (74 - 100)	0.2
**FEV1 (L)**	2.25 (2.01- 2.56)	2.80 (2.35 - 3.14)	0.11
**FEV1 (%)**	92 (79 - 102)	96 (77 - 104)	0.4
**TLC (L)**	3.83 (3.49 - 4.91)	4.65 (4.22 - 5.90)	**0.027**
**TLC (%)**	75 (58 - 80)	82 (67 - 87)	0.068
**DLCO (%)**	59 (49 - 65)	67 (57 - 74)	0.2
Oxygen therapy			
**On effort - n° (%)**	9 (60%)	4 (13%)	**0.002**
**At rest - n° (%)**	6 (40%)	1 (3.2%)	**0.003**
Therapy			
**Immunosoppressive/steroid - n° (%)**	12 (80%)	25 (76%)	0.90
**Antifibrotic - n° (%)**	10 (67%)	0 (0%)	**<0.001**
Radiological features			
**Honeycombing - yes n°**	1 (6.7%)	2 (6.1%)	>0.9
**Reticulations - yes n°**	11 (73%)	21 (64%)	0.5
**Traction bronchiectasis - yes n°**	12 (80%)	9 (27%)	**<0.001**
**Consolidations - yes n°**	2 (13%)	14 (42%)	**0.048**
**Ground glass - yes n°**	5 (33%)	16 (48%)	0.3
Histological features			
**Lymhphoid aggregates – n**	0 (0.00 - 5.50)	0 (0.00 - 1.00)	0.2
**Follicles with germinal centers - n**	1 (6.7%)	0 (0%)	0.3
**Granulomas – n**	0 (0.00 - 0.00)	0 (0.00 - 0.00)	0.2
**Foamy macrophage -yes (%)**	0 (0%)	6 (18%)	0.2
**Pigmented macrophages - yes (%)**	9 (60%)	15 (45%)	0.4
**Increased alveolar macrophages – yes (%)**	12 (80%)	17 (52%)	0.061
Inflammation distribution			
**Patchy - yes (%)**	9 (60%)	20 (61%)	>0.9
**Extensive - yes (%)**	4 (27%)	12 (36%)	0.5
Lymphocyte distribution			
**Interstitial - yes (%)**	12 (80%)	29 (88%)	0.7
**Peribronchial - yes (%)**	1 (6.7%)	5 (15%)	0.6
**Lymphocyte Pattern**	3 (20%)	7 (21%)	>0.9
▪ **Patchy - yes (%)**	7 (47%)	8 (24%)	0.2
▪ **Extensive - yes (%)**	3 (20%)	6 (18%)	>0.9
**Fibroblast foci – n**	0 (0.00 - 0.50)	0 (0.00 - 0.00)	0.5
Fibrosis			
**Organizing pneumonia (%)**	5 (33%)	21 (64%)	0.051
**Fibrosis (%)**	36 (31 - 50)	44 (24 - 57)	0.4
▪ **Any pattern fibrosis - yes (%)**	7 (47%)	6 (18%)	0.077
▪ **Patchy - yes (%)**	2 (13%)	2 (6.1%)	0.6
▪ **Extensive - yes (%)**	5 (33%)	4 (12%)	0.11
Location fibrosis			
**Interstitial - yes (%)**	13 (87%)	27 (82%)	>0.9
**Subpleural - yes (%)**	0 (0%)	3 (9.1%)	0.5
**Centrilobular - yes (%)**	4 (27%)	3 (9.1%)	0.2
Other histological features			
**Microhoneycombing - yes (%)**	3 (20%)	3 (9.1%)	0.4
**SRIF - yes (%)**	1 (6.7%)	0 (0%)	0.3
**Alveolar wall thickening - yes (%)**	2 (13%)	4 (12%)	>0.9
**Thrombi - yes (%)**	1 (6.7%)	0 (0%)	0.3
**Ossification - yes (%)**	0 (0%)	1 (3.0%)	>0.9
**Bronchiolar metaplasia - yes (%)**	6 (40%)	7 (21%)	0.3
**Anthracosis - yes (%)**	8 (53%)	25 (76%)	0.2
**Interstitial SMM - yes (%)**	2 (13%)	3 (9.1%)	0.6
**Enlarged alveoli - yes (%)**	2 (13%)	2 (6.1%)	0.6

To compare demographics between PPF (progressive pulmonary fibrosis) and nPPF (nonprogressive pulmonary fibrosis), the chi-square test and the Fisher’s t-test for categorical variables, and Mann–Whitney t-test for continuous variables were used. SMM, smooth muscle metaplasia; SRIF, smoking related interstitial fibrosis; FVC, Forced Vital Capacity; DLCO, Diffusion Lung CO; TLC, total lung capacity. Values are expressed as numbers and (%) or median and range, as appropriate. Statistical significance is highlighted in bold.

### 3.3 Histological and molecular findings

**Figure 1:**
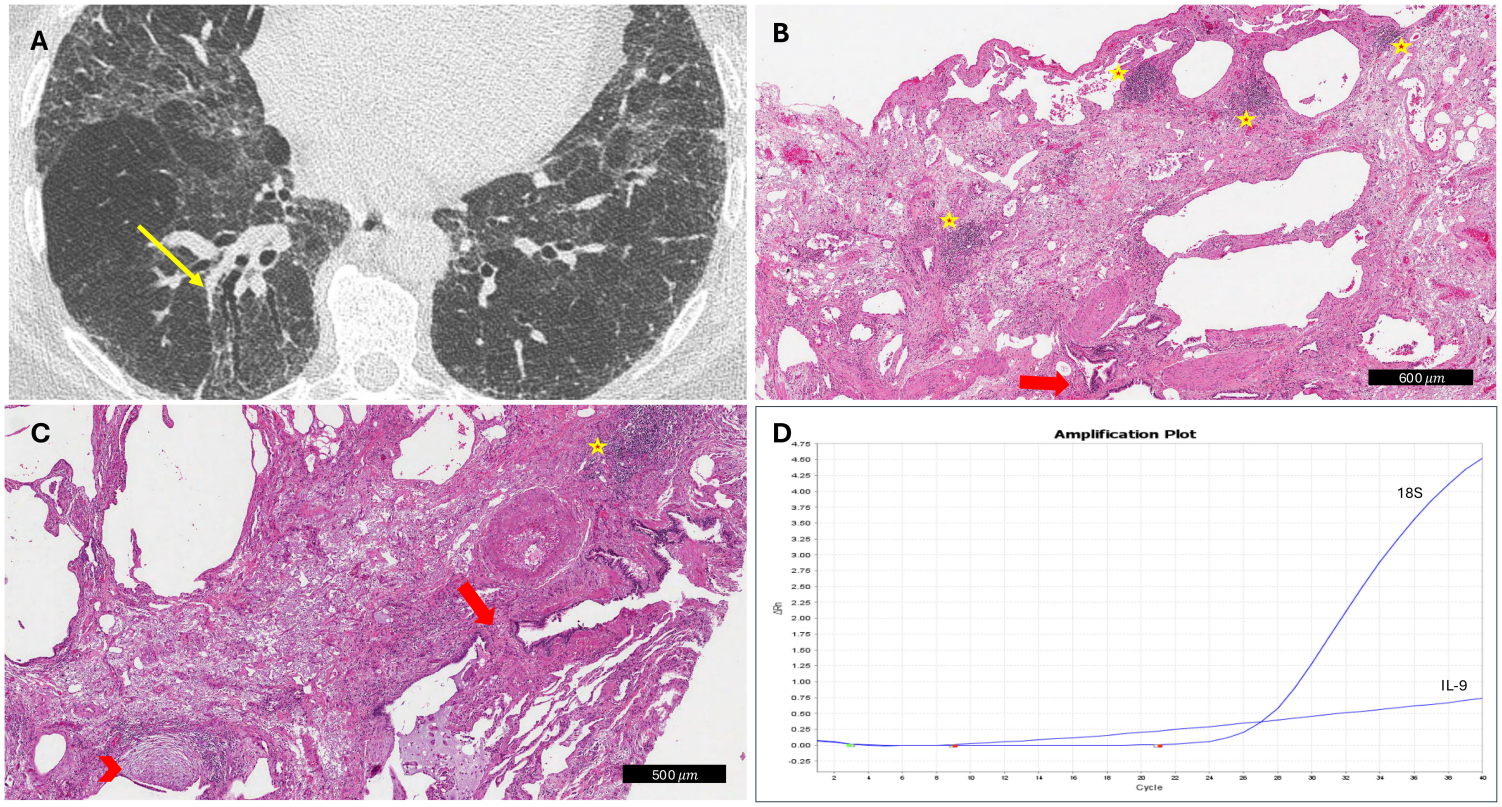
Explanatory case of progressive fibrosing interstitial lung disease. **(A)** CT scan showed traction bronchiectasis at diagnosis (yellow arrow). **(B, C)** At histology, several lymphoid aggregates (yellow stars), traction bronchiectasis (red arrows), and fibroblastic foci (red arrowhead) were detected (hematoxylin and eosin, scale bar: 500 and 600 μm, respectively). **(D)** IL9 overexpression was detected by molecular analyses (IL-9 curve with the inclusion of the internal control gene 18S as a reference).

**Figure 2:**
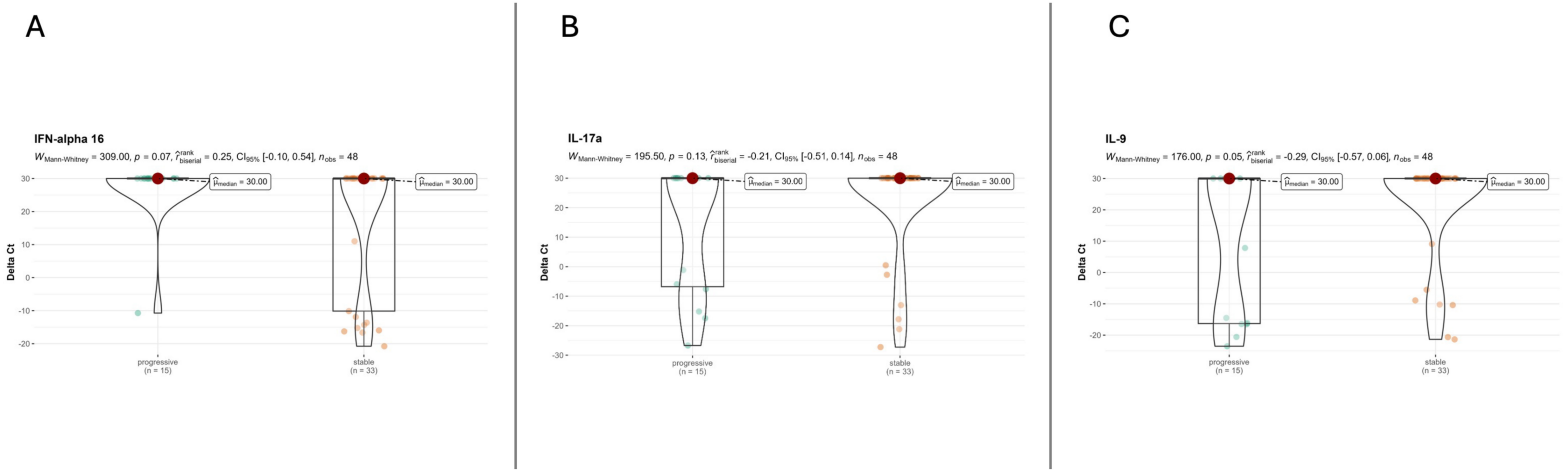
Cytokine expression dividing patients into PPF and nPPF. **(A)** IFN-alpha 16 analysis between PPF and nPPF. **(B)** IL-17a analysis between PPF and nPPF. IL9 analysis between PPF and nPPF **(C)**.

The histological analysis did not yield statistically significant results but revealed significant gradients for some variables. Even if an increased level of alveolar macrophages was detected in lung specimens of patients with PPF (80% vs. 52%; p=0.061), organizing pneumonia (%) seemed to be more prevalent in patients with nPPF (33% vs. 64%; p=0.051). Analysis of the severity and distribution of inflammatory cell infiltration (interstitial or peribronchial) showed no differences between PPF and nPPF patients. All detailed histological analyses are reported in **Table 2**. An index case is presented in **Figure 1**. All cytokine analyses are reported in the supplement data, **Supplementary Table S3**. These histological trends were further supported by qPCR findings, which showed a statistically significant difference in IL9 expression (p=0.049) between PPF and nPPF patients, as well as near-significant differences in IL17a and IFN-alpha 16 expression levels (p=0.06 and p=0.07, respectively), as reported in **Figure 2**.

### 3.4 Boruta analysis algorithm and regression analyses

**Figure 3:**
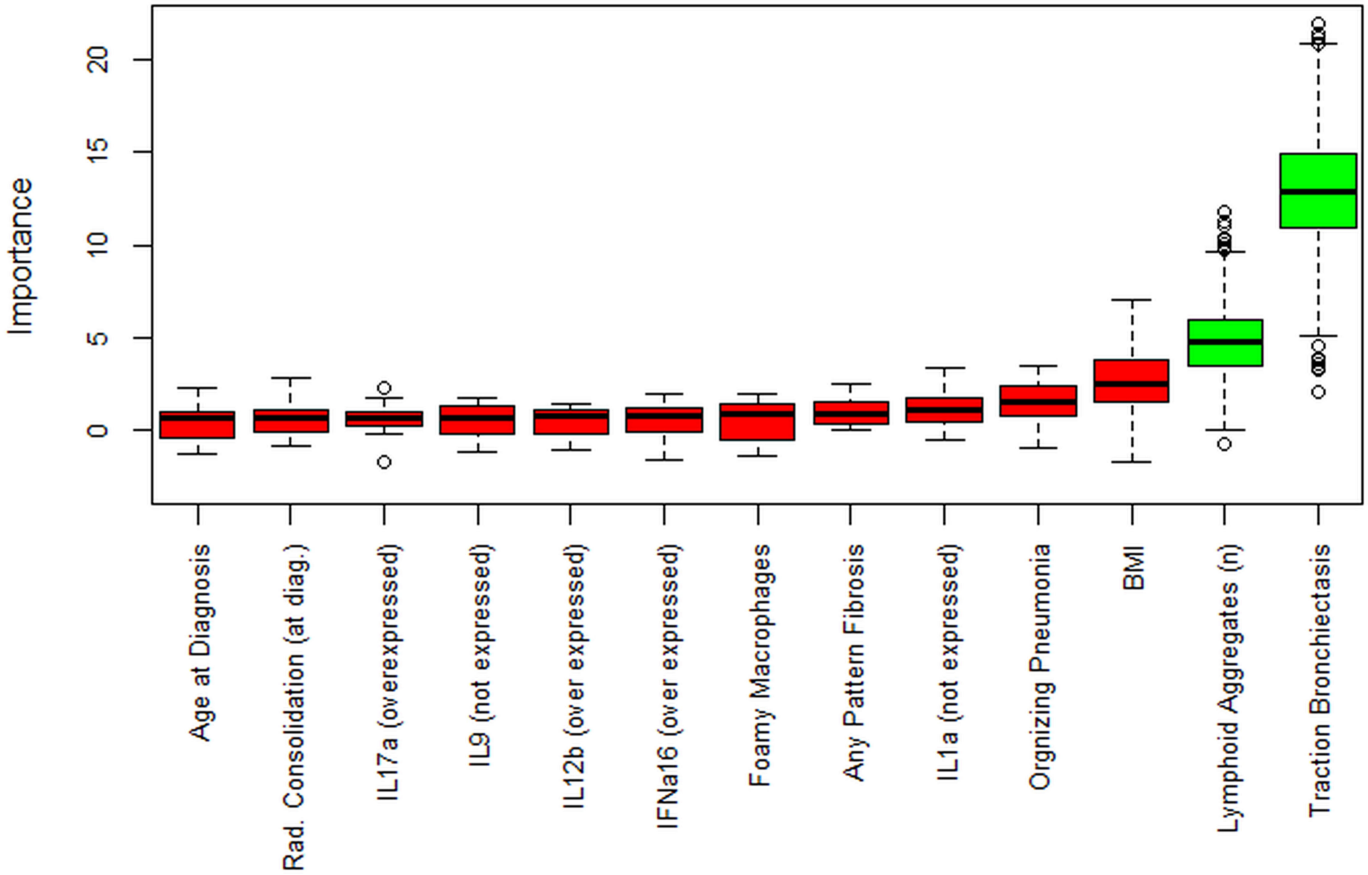
Boruta analysis. Boruta feature selection shows predictive significance for a higher number of lymphoid aggregates and traction bronchiectasis (in green). Unfilled circles indicate outliers. Not all the initial variables are shown. For the full list of variables, see the code in the linked repository.

The Boruta analysis algorithm was used to detect relevant predictors that impact the outcome of interest (i.e., the progressive phenotype). This analysis showed that the two most important variables in determining the PPF status are the number of lymphoid aggregates and the presence of traction bronchiectasis (**Figure 3**). Logistic regression was also performed to evaluate independent predictors of disease progression. In the analysis we considered traction bronchiectasis at diagnosis, number of lymphoid aggregates, IL9 expression, FVC (%) predicted at diagnosis, and IFNalfa16 expression, and consolidation. At the end, independent predictors of progression were traction bronchiectasis [OR = 6.59; 95CI (1.83 – 94.1); p=0.016] (**Supplementary Table S4**).

## 4 Discussion

PPF, a recently described phenotype in patients with ILD, poses a challenge in clinical practice since the reliable predictors of its progression remain elusive. In this study, we used a comprehensive morphological and molecular cytokine analysis, along with radiological and clinical data, to identify potential markers predictive of a progressive phenotype. Specifically, in our cohort study with a variety of established fibrosing lung diseases, we showed that the PPF group displayed greater functional impairment, a significant prevalence of bronchiectasis at first HRCT, and different inflammatory tissue features and cytokine profile compared to nPPF. Respiratory function tests at diagnosis showed differences between the two groups; indeed, progressive patients displayed lower FVC and TLC values than non-progressive patients. These observations substantially support the role of low lung function as one of the main risk factors predicting disease progression, as previously reported (15, 16).

In the clinical trial setting and practical guidelines, various combinations of increasing respiratory symptoms, reductions in lung function and/or signs of increasing fibrosis on HRCT scans have been reported as key predictive markers to take into consideration for the progressive evolution of PPF. However, in patients with PPF who display a heterogeneous and variable course, the validation of a scoring system to predict progression continues to be a great challenge. CT features at first observation, including the presence of a UIP pattern have consistently predicted a higher likelihood of progression. Whereas in patients with IPF several studies have highlighted the importance of traction bronchiectasis as a key CT feature associated with higher mortality risk (17, 18). On multivariate analysis of CT patterns, the severity of traction bronchiectasis, in particular, was superior to pulmonary function tests for predicting mortality in 92 patients with chronic hypersensitivity pneumonitis (19). Moreover, traction bronchiectasis was noted within interstitial lung abnormalities (ILA) on CT, which is often associated with poor survival in recent studies and probably considered an earlier sign of fibrosing lung disease in those patients (20). In our study, PPF patients showed a significantly higher prevalence of traction bronchiectasis at first HRCT. This was a key finding as it was then confirmed in the Boruta analysis as one of the most important predictive markers of disease progression despite therapy. Since the occurrence of traction bronchiectasis is easy to verify, it should always be reported and, above all, implemented in the radiology report at the initial clinical evaluation to provide useful information for appropriate prognostic stratification. Of course, a longitudinal radiologic evaluation and scoring system would be the next desirable step.

Conversely, it is well-documented that the definitive UIP pattern serves as a significant indicator of disease progression (21, 22). Within our cohort, only three patients exhibited honeycombing at the time of diagnosis. As previously stated, our study deliberately excluded patients diagnosed with IPF. Additionally, we conducted evaluations of patients at the time of diagnosis, which may have contributed to the comparatively low number of patients demonstrating honeycombing. The infrequent occurrence of UIP patterns may elucidate the absence of significant findings observed in the analysis.

In a systematic investigation of the lung parenchyma, we assessed the tissue cytokine profile and found a higher number of lymphoid follicles and a peculiar inflammatory cytokine profile with IL9 overexpressed in PPF compared to nPPF. IL9, a cytokine and growth factor that induces Th2 immune responses, has recently been implicated in several fibrosing/inflammatory lung diseases. It is produced primarily by helper T lymphocytes (Th9 cells) and signals via a receptor expressed on mast cells, macrophages, and T lymphocytes. Moreover, IL9 stimulates B lymphocytes to produce immunoglobulins, which are essential for developing immune memory. It also promotes the proliferation and survival of B cells (23, 24). In the cytokine environment, IL9 is key to forming lymphoid follicles by creating a supportive setting for B cell activation and differentiation. When B cells are activated by IL9, they proliferate, differentiate into plasma cells and memory B cells, and migrate to germinal centers within lymphoid follicles for further maturation and antibody production (25). IL9 also indirectly influences lymphoid follicles by promoting the differentiation of follicular helper T cells, which are vital for forming and maintaining germinal centers. These cells provide necessary signals for B cell maturation and antibody affinity maturation. The link between IL9 and lymphoid follicle formation involves IL9 binding to its receptor on B cells, triggering downstream signaling events that enhance B cell activation, proliferation, and survival. This process contributes to the formation of germinal centers within lymphoid follicles, where critical processes like antibody affinity maturation and establishing long-term humoral immunity occur (26). In a recent study conducted by Deng, K.M., the role of IL9 was deeply investigated in idiopathic pulmonary fibrosis (IPF). The authors found that Th9 cells promote fibroblast differentiation, activation, and collagen secretion by secreting IL9. Moreover, the authors also reported that neutralizing IL9 in both preventive and therapeutic settings ameliorates bleomycin-induced pulmonary fibrosis in their mice model (27). Intriguingly, IL9 seems higher in patients with acute exacerbation of the disease (AE-IPF) in comparison with stable IPF patients, as reported by Weng D. et al. (28), suggesting a critical role of this cytokine in patients with more aggressive conditions. Thus, we can speculate that, at diagnosis, the histologic marker of lymphoid aggregates (LA), together with increased levels of tissue IL9 may help clinicians to better personalize pharmacological treatment and short-term surveillance for patients with fibrosing interstitial lung diseases. It is also notable that experimental test studies have proved the efficacy of a monoclonal antibody (mAb) to IL9 to improve inflammation and fibrosis through a reduction of the levels of Th1 and Th2 cytokines (29).

In addition to IL-9, two other cytokines, IL-17A and IFN-α16, showed a trend toward differential expression between the two groups. IL-17A, a cytokine produced predominantly by Th17 cells, has been implicated in promoting fibroblast activation, neutrophilic inflammation, and extracellular matrix remodeling in experimental models of pulmonary fibrosis (30, 31). Elevated levels of IL-17A have also been reported in patients with IPF and systemic sclerosis-associated ILD, suggesting its involvement in chronic inflammatory circuits that contribute to fibrotic progression (32). Within the present study, the observed trend aligns with these findings and suggests that IL-17A may contribute to the pro-fibrotic milieu in a subset of patients with progressive disease.

Similarly, IFN-alpha16 belongs to the type I interferon family, whose aberrant expression has been associated with epithelial injury, immune dysregulation, and fibrosis in various autoimmune and ILD (33, 34). A sustained type I IFN signature has been described in dermatomyositis- and lupus-associated ILDs and may characterize a subset of fibrosing ILD with autoimmune features (35, 36). The trend toward increased IFN-alpha16 in our cohort may therefore point to an underlying interferon-driven endotype among patients with progressive fibrosing ILD.

### 4.1 Limitations of the study

The present research study is limited by the relatively small sample size and the retrospective single-center study design. However, we recruited a well-characterized population using uniform study methods and robust statistical methodologies, and evaluations were made on tissue samples rather than only blood or BAL, enhancing the reliability of our findings. On the other hand, a strength of our study is the fact that all our patients were enrolled at the moment of diagnosis, thus all these patients were naïve from any kind of immunosuppressive and/or corticosteroid therapy. In this line, we excluded also CTD-ILD patients because some can develop respiratory involvement after the principal rheumatological diagnosis, and thus being in immunosuppressive therapy before the ILD diagnosis.

Finally, we acknowledge that other cytokines, including those overexpressed in the PPF group, may play a relevant biological role; however, due to tissue limitations, protein-level validation was not feasible. Ongoing proteomic analyses are expected to clarify the significance of these findings and complement the current data.

## 5 Conclusions

To the best of our knowledge, this represents the first study examining patients with fibrosing ILD, indicating that a comprehensive analysis including radiological and pathological features, along with a cytokine molecular profile at baseline, may aid in predicting the occurrence of progression despite therapy. Future studies are warranted to substantiate our findings.

## Data Availability

The datasets presented in this study can be found in online repositories. The names of the repository/repositories and accession number(s) can be found below: https://researchdata.cab.unipd.it/id/eprint/1329.
