# Supplementary material for: Predicting biomarkers of progressive pulmonary fibrosis: morphological, cytokine profile, and clinical portrait
Source: Front Immunol. 2025 Jun 19;16:1514439. doi: 10.3389/fimmu.2025.1514439 (PMC12221905; doi:10.3389/fimmu.2025.1514439)
Supplement: Supplementary file 1 [file DataSheet1.doc]

**Title: Predicting biomarkers of progressive pulmonary fibrosis: morphological, cytokine profile, and clinical portrait.**

Authors: Nicol Bernardinello^1^*, Federica Pezzuto^2^*, Lauren D’Sa^3^, Luca Vedovelli^2^, Chiara Giraudo^2^, Anamaria Chelu^1,4^, Cecilia de Chellis^2^, Francesca Lunardi^2^, Francesco Fortarezza^2^, Francesca Boscaro^1^, Elisabetta Cocconcelli^1^, Paolo Spagnolo^1^, Elisabetta Balestro^§1^, Fiorella Calabrese^2†§^.

^1^Respiratory disease unit, Department of Cardiac, Thoracic, Vascular Sciences and Public Health, University of Padova, Padova, Italy.

^2^ Department of Cardia, Thoracic, Vascular Science, and Public Health, University of Padova, Padova 35121, Italy.

^3^ Department of Histopathology, Royal Brompton and Harefield Hospitals, Guy’s and St Thomas’ NHS Foundation Trust, London, UK.

^4^Ospedale Arco di Trento, Trento, Italy

*Nicol Bernardinello and Federica Pezzuto contributed equally to this work as first authors.

§Fiorella Calabrese and Elisabetta Balestro contributed equally to this work as senior authors.

†Corresponding author: [fiorella.calabrese@unipd.it](mailto:fiorella.calabrese@unipd.it)

**SUPPLEMENT MATERIAL**

**Table S1: different diagnoses in patients with PPF and nPPF (n=48).**

|  | **PPF (15)** | **nPPF (33)** |
| --- | --- | --- |
| **fOP** | 2 (13%) | 15 (45%) |
| **fHP** | 5 (33%) | 4 (12%) |
| **NSIP** | 4 (27%) | 4 (27%) |
| **Smoking related ILD** | 3 (20%) | 4 (12%) |
| **Unclassifiable** | 1 (6.7%) | 4 (12%) |
| **PPFE** | 0 (0%) | 2 (6.1%) |
|  | **p=0.113** | |

PPF (progressive pulmonary fibrosis), nPPF (nonprogressive pulmonary fibrosis), fOP: fibrosing organizing pneumonia, fHP: fibrosing hypersensitivity pneumonia, NSIP: non-specific interstitial pneumonia; ILD: interstitial lung disease, PPFE: pleuro parenchymal fibroelastosis.

**Table S2: functional characteristics of patients with PPF and nPPF at last follow up visit.**

|  | **PF-ILD (15)** | **NPF-ILD (33)** | **p-value** |
| --- | --- | --- | --- |
| - **Functional parameters** |  |  |  |
| **FVC (L)** | 2.11 (1.66 -2.44) | 3.64 (2.62 - 3.95) | **<0.0001** |
| **FVC (%)** | 68 (49 -78) | 93 (86 -102) | **<0.0001** |
| **FEV1 (L)** | 1.62 (1.43 - 2.05) | 2.79 (2.24 - 3.14) | **0.002** |
| **FEV1 (%)** | 72 (54 - 83) | 96 (83 - 106) | **0.006** |
| **TLC (L)** | 3.17 (2.69, 3.54) | 4.95 (4.02 - 5.68) | **<0.0001** |
| **TLC (%)** | 55 (48 - 66) | 77 (71 - 84) | **0.0001** |
| **DLCO (%)** | 33 (31 - 52) | 63 (53 - 70) | **<0.0001** |

FVC: Forced Vital Capacity, DLCO: Diffusion Lung CO, TLC: total lung capacity. Values are expressed as numbers and (%) or median and range, as appropriate. To compare demographics between PPF (progressive pulmonary fibrosis) and nPPF (nonprogressive pulmonary fibrosis), the Mann–Whitney t-test for continuous variables was used.

**Table S3: Inflammatory mediator profiles in patients with PPF (progressive pulmonary fibrosis) and nPPF (non progressive pulmonary fibrosis).**

|  | **PF-ILD (15)** | **NPF-ILD (33)** | **p-value** |
| --- | --- | --- | --- |
| **IFN-α1** | 30 (30 - 30) | 30 (30 - 30) | 0.2 |
| Over expressed - yes (%) | 0 (0%) | 3 (9.1%) | 0.5 |
| Not expressed - yes (%) | 14 (93%) | 26 (79%) | 0.4 |
| **IFN-α16** | 30 (30 - 30) | 30 (-10 - 30) | 0.065 |
| Over expressed - yes (%) | 1 (6.7%) | 9 (27%) | 0.14 |
| Not expressed - yes (%) | 14 (93%) | 24 (73%) | 0.14 |
| **IFN-α17** | 30 (-9 - 30) | 14 (-13 - 30) | 0.5 |
| Over expressed - yes (%) | 5 (33%) | 13 (39%) | 0.7 |
| Not expressed - yes (%) | 9 (60%) | 16 (48%) | 0.5 |
| **IFN-α2** | 30 (8 - 30) | 30 (30 - 30) | 0.3 |
| Over expressed - yes (%) | 3 (20%) | 3 (9.1%) | 0.4 |
| Not expressed - yes (%) | 9 (60%) | 25 (76%) | 0.3 |
| **IFN-α6** | 30 (30 - 30) | 30 (9 - 30) | 0.4 |
| Over expressed - yes (%) | 2 (13%) | 5 (15%) | >0.9 |
| Not expressed - yes (%) | 12 (80%) | 22 (67%) | 0.5 |
| **IFN-α7** | 30 (18 - 30) | 30 (5 - 30) | 0.5 |
| Over expressed - yes (%) | 3 (20%) | 6 (18%) | >0.9 |
| Not expressed - yes (%) | 11 (73%) | 21 (64%) | 0.5 |
| **IFN-α8** | 30 (10 - 30) | 30 (8 - 30) | 0.7 |
| Over expressed - yes (%) | 3 (20%) | 6 (18%) | >0.9 |
| Not expressed - yes (%) | 10 (67%) | 20 (61%) | 0.7 |
| **IFN-β1** | 30 (21 - 30) | 30 (13 - 30) | >0.9 |
| Over expressed - yes (%) | 0 (0%) | 4 (12%) | 0.3 |
| Not expressed - yes (%) | 11 (73%) | 24 (73%) | >0.9 |
| **IFN-γ** | 30 (30 - 30) | 30 (30 - 30) | 0.2 |
| Over expressed - yes (%) | 1 (6.7%) | 5 (15%) | 0.6 |
| Not expressed - yes (%) | 14 (93%) | 26 (79%) | 0.4 |
| **IL-10** | 30 (21 - 30) | 30 (10 - 30) | 0.5 |
| Over expressed - yes (%) | 2 (13%) | 7 (21%) | 0.7 |
| Not expressed - yes (%) | 11 (73%) | 21 (64%) | 0.5 |
| **IL-12α** | 30 (30 - 30) | 30 (30 - 30) | 0.3 |
| Over expressed - yes (%) | 0 (0%) | 0 (0%) | >0.9 |
| Not expressed - yes (%) | 14 (93%) | 27 (82%) | 0.4 |
| **IL-12β** | 30 (30 - 30) | 30 (-11 - 30) | 0.2 |
| Over expressed - yes (%) | 2 (13%) | 10 (30%) | 0.3 |
| Not expressed - yes (%) | 13 (87%) | 23 (70%) | 0.3 |
| **IL-13** | 30 (30 - 30) | 30 (30 - 30) | 0.6 |
| Over expressed - yes (%) | 1 (6.7%) | 3 (9.1%) | >0.9 |
| Not expressed - yes (%) | 14 (93%) | 29 (88%) | >0.9 |
| **IL-15** | 30 (3 - 30) | 30 (13 - 30) | 0.9 |
| Over expressed - yes (%) | 4 (27%) | 7 (21%) | 0.7 |
| Not expressed - yes (%) | 10 (67%) | 24 (73%) | 0.7 |
| **IL-16** | 13 (10 - 30) | 10 (8 - 30) | 0.4 |
| Over expressed - yes (%) | 2 (13%) | 5 (15%) | >0.9 |
| Not expressed - yes (%) | 7 (47%) | 12 (36%) | 0.5 |
| **IL-17α** | 30 (-7 - 30) | 30 (30 - 30) | 0.13 |
| Over expressed - yes (%) | 6 (40%) | 5 (15%) | 0.074 |
| Not expressed - yes (%) | 9 (60%) | 27 (82%) | 0.2 |
| **IL18** | 30 (22 - 30) | 30 (15 - 30) | >0.9 |
| Over expressed - yes (%) | 3 (20%) | 4 (12%) | 0.7 |
| Not expressed - yes (%) | 11 (73%) | 24 (73%) | >0.9 |
| **IL1-A** | 30 (8 - 30) | 30 (30 - 30) | 0.2 |
| Over expressed - yes (%) | 2 (13%) | 3 (9.1%) | 0.6 |
| Not expressed - yes (%) | 9 (60%) | 25 (81%) | 0.2 |
| **IL-1β** | 30 (6 - 30) | 30 (8 - 30) | 0.7 |
| Over expressed - yes (%) | 1 (6.7%) | 5 (15%) | 0.6 |
| Not expressed - yes (%) | 8 (53%) | 20 (61%) | 0.6 |
| **IL-2** | 30 (30 - 30) | 30 (30 - 30) | 0.2 |
| Over expressed - yes (%) | 2 (13%) | 1 (3.0%) | 0.2 |
| Not expressed - yes (%) | 13 (87%) | 32 (97%) | 0.2 |
| **IL-3** | 30 (30 - 30) | 30 (30 - 30) | >0.9 |
| Over expressed - yes (%) | 3 (20%) | 7 (21%) | >0.9 |
| Not expressed - yes (%) | 12 (80%) | 26 (79%) | >0.9 |
| **IL-4** | 30 (30 - 30) | 30 (30 - 30) | 0.2 |
| Over expressed - yes (%) | 0 (0%) | 4 (12%) | 0.3 |
| Not expressed - yes (%) | 15 (100%) | 29 (88%) | 0.3 |
| **IL-5** | 30 (30 - 30) | 30 (30 - 30) | 0.4 |
| Over expressed - yes (%) | 0 (0%) | 2 (6.1%) | >0.9 |
| Not expressed - yes (%) | 15 (100%) | 31 (94%) | >0.9 |
| **IL-6** | 30 (6 - 30) | 30 (2 - 30) | 0.5 |
| Over expressed - yes (%) | 3 (20%) | 8 (24%) | >0.9 |
| Not expressed - yes (%) | 9 (60%) | 17 (52%) | 0.6 |
| **IL-8** | 10 (2 - 30) | 30 (8 - 30) | 0.5 |
| Over expressed - yes (%) | 3 (20%) | 4 (12%) | 0.7 |
| Not expressed - yes (%) | 7 (47%) | 18 (55%) | 0.6 |
| **IL-9** | 30 (-16 - 30) | 30 (30 - 30) | **0.049** |
| Over expressed - yes (%) | 6 (40%) | 6 (18%) | 0.2 |
| Not expressed - yes (%) | 8 (53%) | 26 (79%) | 0.094 |
| **lta** | 30 (-10, 30) | 30 (30, 30) | 0.2 |
| Over expressed - yes (%) | 6 (40%) | 6 (18%) | 0.2 |
| Not expressed - yes (%) | 9 (60%) | 26 (79%) | 0.3 |
| **TNF** | 11 (1 - 30) | 30 (9 - 30) | 0.3 |
| Over expressed - yes (%) | 4 (27%) | 4 (12%) | 0.2 |
| Not expressed - yes (%) | 6 (40%) | 18 (55%) | 0.4 |

Data are presented as median (IQR) ΔCt

PPF (progressive pulmonary fibrosis); nPPF (nonprogressive pulmonary fibrosis), IC immunocompromised, IFN interferon, IL interleukin, LTA lymphotoxin-alpha, TNF tumor necrosis factor.

Inflammatory mediators with significant differences among the groups are marked in bold.

**Table S4: logistic regression analysis considering possible predictors of progressive pulmonary fibrosis (PPF).**

| **Characteristic** | **OR** | **95% CI** | **p-value** |
| --- | --- | --- | --- |
| Traction bronchiectasis at diagnosis- yes | 6.59 | 1.83 - 49.1 | **0.016** |
| Lymphoid aggregates - n° | 2.72 | 0.71 - 17.9 | 0.2 |
| IL9 expression | 0.67 | 0.15 - 2.3 | 0.5 |
| FVC(%)predict at diagnosis | 0.17 | 0.01 - 0.76 | 0.06 |
| IFNalfa16 expression | 3.22 | 1.03 - 16.4 | 0.08 |
| Consolidation - yes | 1.76 | 0.43 - 12.1 | 0.5 |
| OR = Odds Ratio, CI = Confidence Interval | | | |

**Figure S1: Figure 1: Study flow-chart**

**
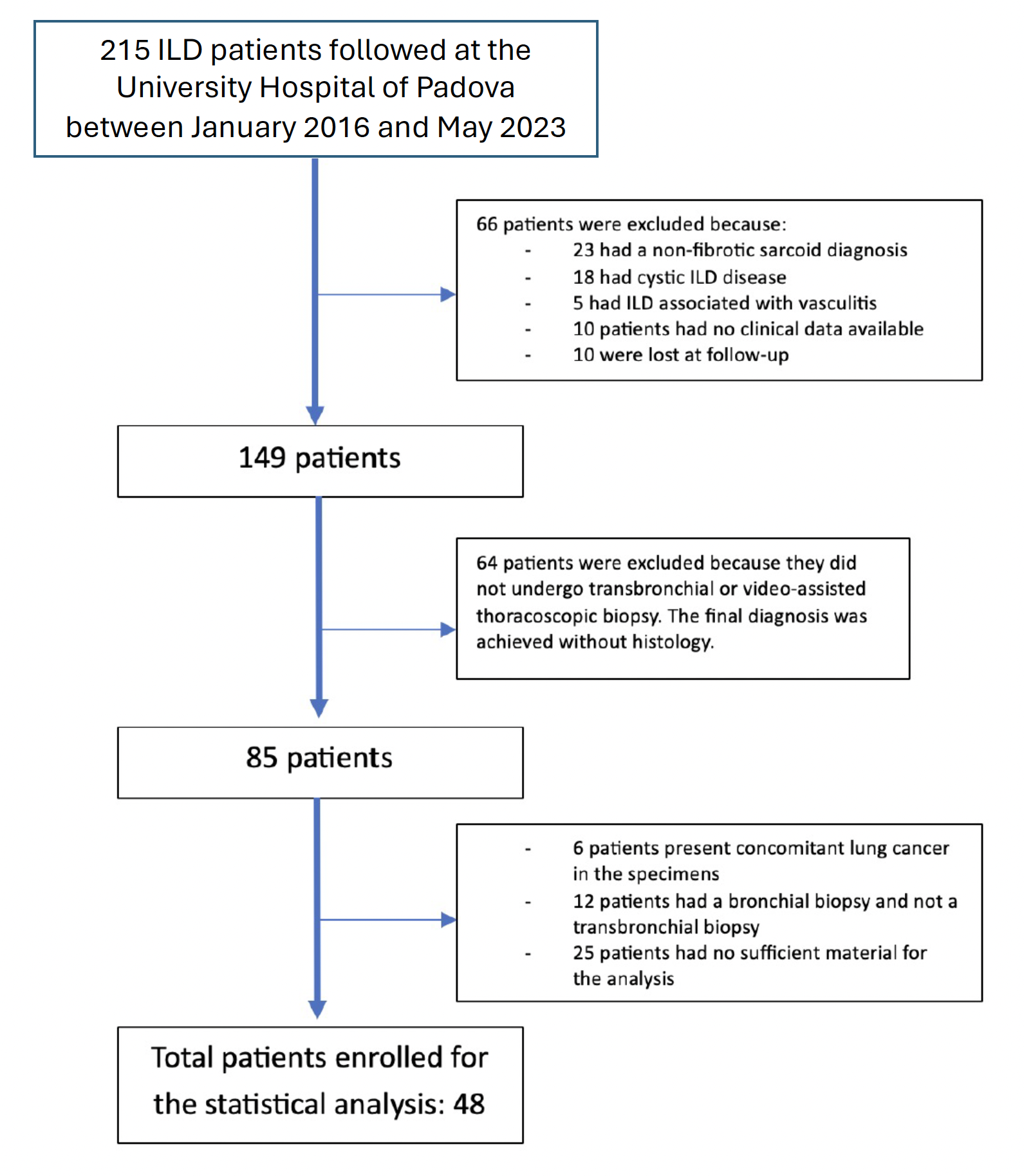
**

**Legend:** ILDs, interstitial lung disease.

**Figure S2: Survival analysis between PPF and nPPF**

Kaplan-Meier was used for survival analysis [p=0.027].
